# Multimodal Quantitative MRI of White and Gray Matter: Associations with Myelin-Related Metrics in the Healthy Adult Brain Using Synthetic MRI and IVIM-DWI

**DOI:** 10.3390/jcm15114240

**Published:** 2026-05-30

**Authors:** Sami A. Alghamdi, Othman I. Alomair, Abdullah H. Abujamea

**Affiliations:** 1 Radiological Sciences Department, College of Applied Medical Sciences, King Saud University, P.O. Box 4545, Riyadh 145111, Saudi Arabia; oalomir@ksu.edu.sa; 2 Medical Physics Section, King Saud University Medical City, King Saud University, P.O. Box 4545, Riyadh 145111, Saudi Arabia; abujamea@ksu.edu.sa

**Keywords:** MRI, myelin imaging, white matter, gray matter, IVIM-DWI, synthetic MRI

## Abstract

**Background/Objectives**: Quantitative MRI may support non-invasive characterization of myelin-related tissue properties, but the relative association of synthetic MRI (SyMRI) metrics and IVIM-DWI parameters with myelin-related measures remains unclear. This study investigated associations between SyMRI-derived quantitative measures, IVIM-derived parameters, and myelin-related indices in healthy adult brain tissue. **Methods**: Twenty-six healthy adults underwent IVIM-DWI and SyMRI. ROI-based quantitative parameters were extracted, including ADC, *D*, D*, perfusion fraction (*f*), T_1_, T_2_, and proton density (PD). SyMRI-derived myelin-correlated parenchymal fraction (MyCPF) and myelin-correlated volume (MyC) were obtained. ROI-averaged WM–GM differences were assessed using the Wilcoxon signed-rank test, and associations were evaluated using Spearman correlation with false discovery rate correction. **Results**: IVIM-derived parameters were significantly higher in WM, whereas T_1_ and PD were significantly higher in GM (all *p* < 0.001); T_2_ did not differ significantly (*p* = 0.901). In WM, PD showed a strong inverse association with MyCPF (ρ = −0.736, *p* < 0.001, q < 0.001), whereas its association with MyC did not remain significant after correction. In GM, PD showed a moderate inverse association with MyCPF (ρ = −0.532, *p* = 0.005), but this was not significant after correction (q = 0.073). No IVIM-derived parameter showed significant associations with myelin-related metrics after correction. **Conclusions**: PD showed the strongest association with MyCPF, particularly in WM, supporting its potential as a non-invasive marker of myelin-related tissue composition. IVIM-derived parameters showed limited relevance for myelin assessment in this healthy cohort.

## 1. Introduction

Magnetic resonance imaging (MRI) plays a pivotal role in characterizing the structural and microstructural features of brain tissue, particularly in the evaluation of gray matter (GM) and white matter (WM) [1,2]. GM consists mainly of neuronal cell bodies and is essential for higher-order cognitive processing, whereas WM is composed of myelinated axonal fibers that facilitate efficient neural signal transmission across distributed brain networks. The integrity of WM is strongly influenced by its myelin content, which is a key determinant of conduction velocity and overall brain connectivity. The preservation of both GM and WM is therefore essential for normal neurocognitive function, and advanced MRI modalities have been increasingly utilized to characterize their distinct biological properties [3,4].

Quantitative MRI techniques, such as synthetic MRI (SyMRI) and intravoxel incoherent motion diffusion-weighted imaging (IVIM-DWI), provide objective and reproducible measurements that extend beyond conventional qualitative imaging [5,6,7]. SyMRI enables rapid simultaneous quantification of T_1_, T_2_, and proton density (PD), allowing the generation of multiple contrast-weighted images and quantitative parametric maps from a single acquisition [8,9]. These parameters reflect key tissue properties, including composition, water content, and microstructural heterogeneity [10,11]. In addition, SyMRI supports automated tissue segmentation and enables the derivation of global brain metrics, including myelin-related measures such as the myelin-correlated volume (MyC) and the myelin-correlated parenchymal fraction (MyCPF). MyC reflects the absolute amount of myelin-related tissue, whereas MyCPF represents the proportion of myelin-correlated volume relative to brain parenchymal volume (MyCPF = MyC/BPV), providing a normalized measure of myelin content [5,12,13,14].

IVIM-DWI extends conventional diffusion imaging by applying a bi-exponential model that separates perfusion-related pseudo-diffusion from true molecular diffusion. This approach yields quantitative parameters, including *D*, D*, and *f*, which reflect tissue microstructure and microvascular perfusion characteristics [15,16,17]. These parameters differ between WM and GM due to their distinct vascular and microstructural properties, with GM typically exhibiting higher perfusion fractions. Moreover, IVIM enables the assessment of microvascular perfusion without the use of contrast agents, which is particularly advantageous in vulnerable patient populations [18]. However, IVIM-derived parameters primarily reflect diffusion and microvascular perfusion, and their relationship with myelin-related tissue properties remains uncertain.

Previous studies applying each modality independently have demonstrated promising results. SyMRI has been used to quantify myelin-related measures and perform brain tissue segmentation [19,20], while IVIM has shown sensitivity to microvascular and microstructural alterations in both normal and diseased brain tissue [6,15,16]. Despite these advances, studies integrating SyMRI and IVIM-DWI remain limited, with most investigations focusing on single-modality approaches or combining SyMRI with conventional mono-exponential diffusion metrics such as ADC. For example, Zhang et al. [21] reported improved diagnostic performance when combining SyMRI parameters with diffusion imaging in nasopharyngeal tumors. However, conventional DWI does not capture perfusion-related effects, whereas IVIM provides a more comprehensive characterization of tissue microstructure through its bi-exponential model.

The complementary relationship between SyMRI-derived myelin-related metrics and IVIM-derived diffusion/perfusion parameters remains insufficiently defined, particularly when both modalities are evaluated within the same cohort and anatomical regions. This gap limits the biological interpretation of multimodal quantitative MRI parameters and provides the rationale for the present cross-modality analysis. Therefore, this study was planned with the goal of investigating the correlations between IVIM-DWI parameters and SyMRI-derived quantitative measures in healthy adult WM and GM, with a specific focus on their association with SyMRI-derived myelin-related metrics (MyCPF and MyC). The ultimate aim is to establish baseline cross-modality relationships for future neuropathological studies.

## 2. Materials and Methods

### 2.1. Participants

A total of 28 healthy adult participants were initially considered for inclusion in this study. Two subjects were excluded due to significant imaging artifacts that compromised data quality. The final study cohort thus consisted of 26 healthy controls with structurally normal MRI and analyzable multimodal data. Participants were included if they were adults with structurally normal brain MRI findings and no known history of neurological or psychiatric disorders. Subjects were excluded if they had significant motion degradation, imaging artifacts, or incomplete quantitative MRI data that affected map quality.

Participants ranged in age from 18 to 48 years. All MRI examinations were reviewed by a board-certified neuroradiology consultant and confirmed to be structurally normal, ensuring the absence of visible brain abnormalities.

### 2.2. Ethical Approval

This study was conducted in accordance with the Declaration of Helsinki and approved by the Institutional Review Board of King Saud University Medical City, Riyadh, Saudi Arabia (Project No. E-25-9848; approval date: 30 June 2025). Participants were enrolled between 7 July 2025 and 14 July 2025, after IRB approval had been obtained.

### 2.3. MRI Acquisition Protocol

MRI examinations were performed using a 1.5 Tesla system (GE Optima MR450w; GE Healthcare, Milwaukee, WI, USA) equipped with a 20-channel head and neck coil. The imaging protocol included IVIM-DWI and SyMRI sequences for quantitative brain tissue characterization.

The IVIM-DWI sequence was acquired using a single-shot spin-echo-planar imaging technique with multiple b-values (0, 30, 50, 70, 100, 200, 500, and 1000 s/mm^2^). This range of b-values was selected to enable reliable separation of diffusion and perfusion components for IVIM parameter estimation, as described in previous studies [15,22,23].

Synthetic MRI data were acquired using the MAGiC sequence, enabling simultaneous quantification of T_1_, T_2_, and PD maps from a single acquisition. Imaging parameters included a repetition time of 4500 ms, echo time of 26 ms, inversion time of 12.3 ms, matrix size of 320 × 224, field of view of 240 × 240 mm^2^, and slice thickness of 4.5 mm. These quantitative maps provide quantitative tissue characterization and enable extraction of global brain metrics.

### 2.4. Image Registration

To ensure spatial consistency across multimodal datasets, image registration was performed using FSL (FMRIB Software Library, version 6.0.7, Oxford, UK), which provides established tools for MRI image registration and analysis [24,25]. The PD image was selected as the anatomical reference due to its high tissue contrast and reliable delineation of WM and GM structures.

Synthetic MRI maps (T_1_, T_2_, and PD) were inherently co-registered, as they were derived from the same acquisition and shared identical spatial geometry. In contrast, IVIM-derived parametric maps were obtained from a separate diffusion acquisition and exhibited differences in matrix size. Therefore, only IVIM maps were registered to the PD reference space using an affine transformation model implemented in the FLIRT tool. A 12-degrees-of-freedom transformation was applied to account for translation, rotation, scaling, and shear, with trilinear interpolation used during resampling [15].

Following registration, all images were visually inspected to confirm accurate anatomical alignment, particularly within deep GM and WM regions. This approach ensured voxel-wise correspondence across modalities while preserving the quantitative integrity of synthetic MRI maps.

**Figure 1:**
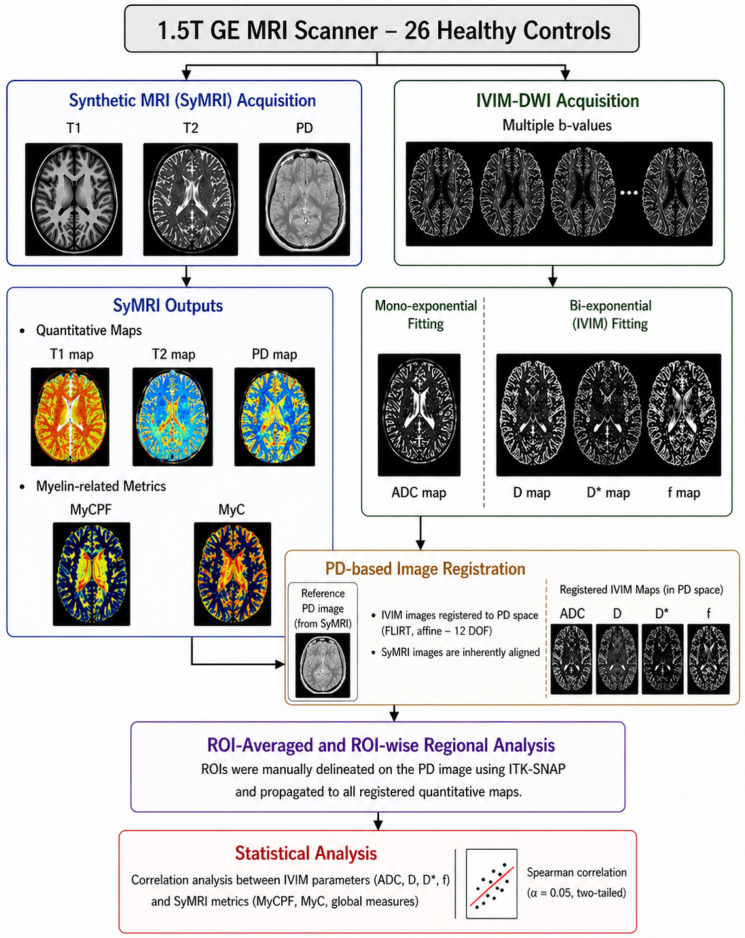
Schematic workflow of the multimodal MRI processing and analysis pipeline used in the present study, integrating SyMRI and IVIM-DWI.

A schematic overview of the complete data processing workflow, including parametric map generation, PD-based registration, ROI definition, and feature extraction, is presented in Figure 1.

### 2.5. IVIM Processing

IVIM data were post-processed to generate quantitative parametric maps reflecting diffusion and perfusion characteristics of brain tissue. The IVIM model is based on bi-exponential signal decay that separates true molecular diffusion from perfusion-related pseudo-diffusion components.

IVIM parameters were extracted using the IB Diffusion™ plug-in (version 21.12; Imaging Biometrics, Elm Grove, WI, USA) integrated into the OsiriX MD platform [22,23]. The segmented IVIM model was employed, with a b-value inflection point set at 200 s/mm^2^. D* was calculated using b-values below this threshold, while true *D* was calculated using b-values above it. The perfusion fraction (*f*) was estimated as the ratio of the perfusion-related signal relative to the total signal decay [15]. In addition, ADC maps were generated using a mono-exponential model.

### 2.6. Synthetic MRI and Global Metrics

Synthetic MRI data were processed using SyMRI software (Version 11.3: SyntheticMR, Linköping, Sweden), which automatically generates quantitative T_1_, T_2_, and PD maps based on multiparametric relaxometry and provides automated tissue segmentation and SyMRI-derived global brain metrics. These included brain parenchymal fraction (BPF), myelin-correlated parenchymal fraction (MyCPF), myelin-correlated volume (MyC), cerebrospinal fluid volume (CSF), brain parenchymal volume (BPV), and intracranial volume (ICV). MyC represents the estimated volume of tissue attributed to the myelin-related component, whereas MyCPF represents the proportion of MyC relative to BPV. These parameters provide quantitative measures of brain tissue composition and global myelin-related characteristics and have been validated in previous studies for assessing brain structure and microstructural integrity [5,19].

### 2.7. ROI Definition and Regional Analysis

Regions of interest (ROIs) were manually delineated on the PD image using ITK-SNAP (version 3.8.0), a validated tool for medical image segmentation and ROI-based analysis [26]. The PD image was selected as the reference for ROI placement due to its high anatomical contrast and clear differentiation between WM and GM structures.

**Figure 2:**
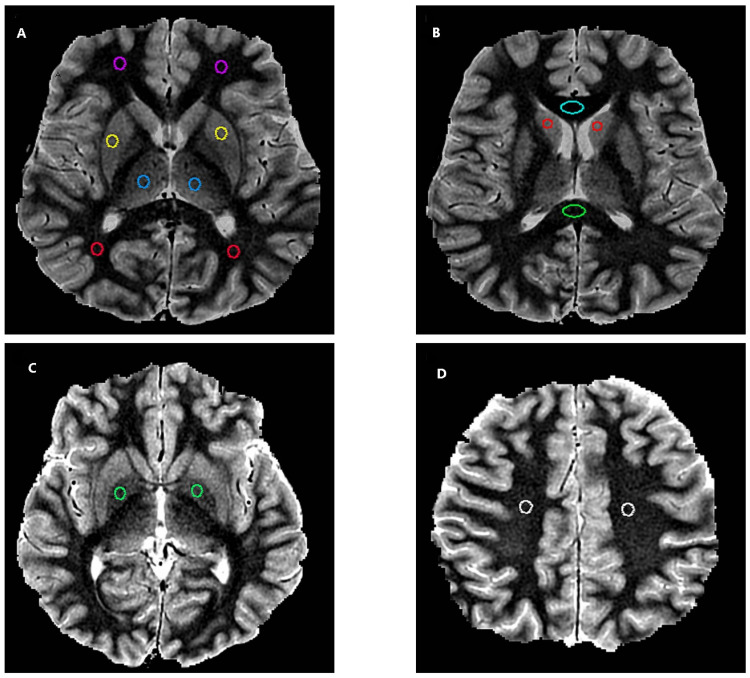
PD images showing the different ROIs. (**A**) Purple represents two ROIs for frontal WM, red represents two ROIs for posterior WM, blue represents two ROIs for the thalamus, and yellow represents ROIs for the putamen regions. (**B**) Red represents two ROIs for the caudate, blue represents the genu, and green represents the splenium. (**C**) Green represents two ROIs for the globus pallidus. (**D**) White represents two ROIs for subcortical WM.

The ROIs defined on the PD image included key regions of interest in both GM and WM. ROIs were placed bilaterally where applicable. Deep GM regions included the putamen, globus pallidus, caudate, and thalamus, while WM regions included the genu and splenium of the corpus callosum, frontal white matter, posterior white matter, and subcortical white matter (Figure 2). To ensure accurate placement, care was taken to avoid contamination from cerebrospinal fluid and vascular structures. Once the ROIs were manually delineated on the PD image, they were directly applied to the inherently aligned SyMRI-derived T_1_, T_2_, and PD maps. The same ROIs were then applied to the IVIM-derived parametric maps after registration to the PD reference image. This approach minimized variability associated with independent ROI placement and improved the reliability of comparisons across modalities.

### 2.8. Statistical Analysis

Statistical analyses were performed using IBM SPSS Statistics (version 26.0; IBM Corp., Armonk, NY, USA). Continuous variables are expressed as mean ± standard deviation (SD). Normality of data distribution was assessed using the Shapiro–Wilk test. Given the mixed distribution of the data and the relatively small sample size, non-parametric statistical methods were applied.

Differences between WM and GM were assessed using the Wilcoxon signed-rank test for paired comparisons within subjects across all quantitative MRI parameters. Effect sizes were additionally calculated using rank-biserial correlation to quantify the magnitude of WM–GM differences beyond statistical significance.

Associations between myelin-related metrics (MyCPF and MyC) and quantitative MRI parameters were evaluated using Spearman’s rank correlation coefficient (ρ). Correlation analyses were performed separately for WM and GM composite values and included both IVIM-derived and SyMRI-derived parameters.

An ROI-wise correlation analysis was also conducted to assess regional associations between PD and MyCPF across selected brain regions. Given the multiple statistical comparisons performed, there is an increased risk of type I error (false-positive findings). To address this, false discovery rate (FDR) correction was applied using the Benjamini–Hochberg method. This correction was applied separately within each analysis family (WM correlations, GM correlations, and ROI-wise analyses). Adjusted *p*-values were reported as q-values, with q < 0.05 considered statistically significant.

All statistical tests were two-tailed, and *p* < 0.05 was considered statistically significant prior to correction.

## 3. Results

**Figure 3:**
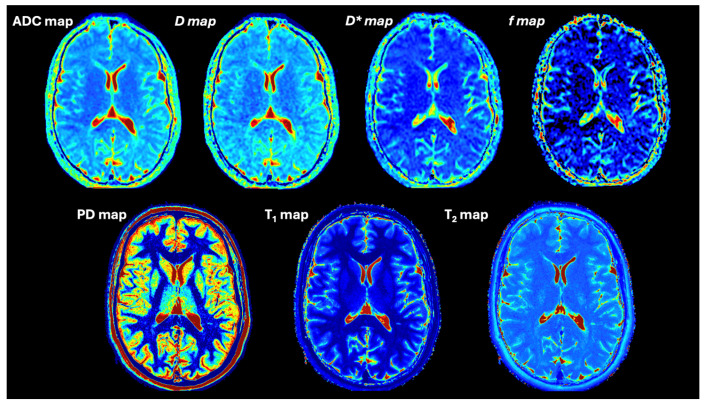
Representative image from one of our samples showing IVIM maps (top row: ADC, *D*, D*, *f*) and synthetic MRI maps (T_1_, T_2_, PD).

The images presented in Figure 3 provide a visual representation of the quantitative MRI parameters used in this study, including both IVIM-derived parameters (ADC, *D*, D*, and *f*) and synthetic MRI maps (T_1_, T_2_, and PD).

### 3.1. Differences Between ROI-Averaged WM and GM Values

Significant differences were observed between WM and GM across most quantitative MRI parameters, based on values averaged across the predefined ROIs for each tissue type. The results of the paired comparisons are presented in Table 1. All IVIM-derived parameters were significantly higher in WM than in GM (all *p* < 0.001), with large effect sizes. In contrast, T_1_ and PD values were significantly higher in GM (*p* < 0.001), also with large effect sizes. No statistically significant difference was observed in T_2_ values between GM and WM (*p* = 0.901), which was associated with a negligible effect size.

### 3.2. Global Myelin and Brain Metrics

SyMRI-derived global brain and myelin-related metrics are presented in Table 2. The mean MyCPF was 13.46 ± 0.95%, while the mean MyC was 183.5 ± 17.89 mL, indicating relatively consistent myelin-related measurements across the healthy cohort. Global volumetric measures also showed expected values for healthy adult brain tissue, including a mean BPV of 1362.69 ± 78.89 mL and a mean ICV of 1554 ± 93.16 mL. The mean BPF was 87.73 ± 2.90%, while the mean CSF volume was 191.35 ± 50.42 mL.

### 3.3. Association Between Myelin Metrics and ROI-Averaged MRI Parameters in WM

This study primarily focused on the relationship between myelin-related metrics and quantitative MRI parameters within WM. Spearman correlation analysis revealed that PD demonstrated the strongest association with myelin metrics, as shown in Table 3. PD in WM exhibited a strong inverse correlation with MyCPF (ρ = −0.736, *p* < 0.001), which remained statistically significant after correction for multiple comparisons (q < 0.001). Although PD also showed a moderate inverse correlation with MyC (ρ = −0.435, *p* = 0.027), this association did not remain significant after FDR correction. No IVIM-derived parameter demonstrated a statistically significant association with myelin metrics after correction for multiple comparisons.

### 3.4. Association Between Myelin Metrics and ROI-Averaged MRI Parameters in GM

In GM, correlations between quantitative MRI parameters and myelin-related metrics were generally weak and inconsistent, as shown in Table 4. Proton density (PD) demonstrated a moderate inverse association with MyCPF (ρ = −0.532, *p* = 0.005); however, this association did not remain statistically significant after correction for multiple comparisons (q = 0.073). No other parameters showed significant correlations with either MyCPF or MyC after FDR correction. Although D* and f exhibited nominal associations with MyC (*p* = 0.029 and *p* = 0.043, respectively), these were no longer significant after adjustment (q > 0.05).

### 3.5. ROI-Wise Analysis of PD–MyCPF Associations

**Figure 4:**
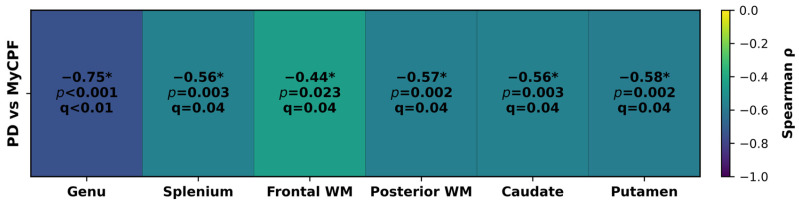
ROI-wise correlations between PD and MyCPF across selected brain regions. Color variation reflects the strength of the inverse association according to Spearman’s ρ. Asterisks denote statistical significance after FDR correction (* q < 0.05).

An ROI-wise correlation analysis was performed to evaluate regional variations in the association between PD and MyCPF across selected brain regions (Figure 4). The strongest inverse association was observed in the genu of the corpus callosum (ρ = −0.748, *p* < 0.001, q < 0.001). Significant inverse associations were also observed in the putamen (ρ = −0.583, *p* = 0.002, q = 0.004), posterior white matter (ρ = −0.569, *p* = 0.002, q = 0.004), caudate (ρ = −0.563, *p* = 0.003, q = 0.004), splenium (ρ = −0.556, *p* = 0.003, q = 0.004), and frontal white matter (ρ = −0.443, *p* = 0.023, q = 0.023). These findings indicate that the inverse association between PD and MyCPF was observed across multiple WM and subcortical GM regions after FDR correction, with the strongest effect identified in the genu.

## 4. Discussion

The present study investigated the relationship between quantitative MRI parameters and myelin-related tissue properties using a multimodal approach integrating SyMRI and IVIM-DWI. The term “myelin-related” refers to SyMRI-derived myelin-correlated metrics rather than direct histological measures of myelin. The key findings include: (i) clear tissue-specific differences between WM and GM, (ii) a strong association between PD and MyCPF in WM but not in ROI-averaged GM after correction, and (iii) no significant associations between IVIM-derived parameters and myelin-related metrics.

The observed differences between WM and GM are consistent with well-established biophysical principles. Importantly, these differences were associated with large effect sizes, indicating that the observed WM versus GM contrasts are not only statistically significant but also biologically meaningful. All IVIM-derived parameters were higher in WM, which may reflect differences in tissue organization and microstructural properties. In contrast, GM exhibited higher PD and T_1_ values, likely due to its higher free water content and lower myelin density. These findings align with prior quantitative MRI studies demonstrating distinct tissue-specific imaging profiles [27].

The most important finding of this study is the strong inverse association between PD and MyCPF in WM, which remained statistically significant after FDR correction. This relationship is biologically plausible because PD is sensitive to mobile proton density, tissue water content, and macromolecular composition. In highly myelinated WM, compact myelin sheaths reduce free water content, resulting in lower PD values and explaining the observed inverse PD–MyCPF relationship. These findings are consistent with prior work showing that SyMRI-based myelin estimation incorporates PD together with relaxation parameters and that PD is related to other myelin-sensitive quantitative MRI techniques [5,28]. In contrast, associations with MyC were weaker and did not remain significant after correction, which may reflect the greater sensitivity of volumetric estimates to spatial sampling and segmentation variability compared with normalized metrics such as MyCPF [8,20,29].

Meanwhile, IVIM-derived parameters did not show significant associations with myelin-related measures. This likely reflects the fundamentally different physiological basis of IVIM metrics, which capture diffusion and microvascular perfusion rather than intrinsic tissue composition and are therefore not expected to directly reflect myelin content, as supported by previous studies [9,30,31,32]. This physiological distinction may explain the absence of significant IVIM–myelin associations in the present study.

Taken together, these findings indicate that the sensitivity of quantitative MRI parameters to myelin-related properties is tissue dependent. The absence of significant associations in ROI-averaged GM after FDR correction may reflect the lower myelin-related contrast and greater regional heterogeneity of GM compared with WM. This interpretation is consistent with previous studies showing that myelin-sensitive imaging techniques generally demonstrate stronger reliability and contrast in WM than in GM [10,33,34].

However, the absence of significant associations at the ROI-averaged GM level does not necessarily indicate the absence of underlying biological relationships. Rather, it may reflect the intrinsic heterogeneity of GM, where averaging multiple anatomically distinct regions can dilute localized microstructural associations. Subcortical GM structures, such as the caudate and putamen, differ from cortical GM in their tissue composition and may exhibit higher myelin-related content and more organized microstructural properties. Recent multiparametric quantitative MRI studies have demonstrated spatial heterogeneity within subcortical nuclei, particularly the putamen, across various biophysical parameters [35]. In addition, synthetic MRI-based quantitative atlases have shown that PD and myelin-related indices provide region-specific information on tissue composition [36]. Therefore, the significant ROI-wise associations observed in the caudate and putamen likely reflect localized PD sensitivity to myelin-related tissue properties that may not be detectable when GM regions are averaged into a single composite measure.

At the regional level, the strongest PD–MyCPF association was observed in the genu of the corpus callosum, while significant inverse PD–MyCPF associations were also observed in the splenium, posterior WM, frontal WM, caudate, and putamen after FDR correction. The prominent association in the genu is consistent with previous studies demonstrating that highly organized WM tracts exhibit increased sensitivity to microstructural variations [32,37,38]. Importantly, the significant associations observed in frontal and posterior WM suggest that the PD–MyCPF relationship is not restricted to commissural fibers but extends across broader WM compartments. Taken together, these ROI-wise findings support the regional specificity of PD–MyCPF associations and emphasize the importance of localized analysis when evaluating myelin-related tissue properties.

From a methodological perspective, these findings underscore the advantage of quantitative MRI over conventional imaging approaches. Unlike conventional MRI, which relies on relative signal intensities, quantitative MRI provides absolute measurements of tissue properties, enabling more objective and reproducible characterization of brain microstructure. This capability is particularly important for developing reliable imaging biomarkers that can be applied across subjects and longitudinal studies [28,39].

The findings of this study have important implications for both research and clinical applications. PD may serve as a useful non-invasive marker of MyCPF-related tissue composition, particularly in WM, whereas IVIM parameters appear to be less suitable for this purpose in healthy brain tissue. Future studies may extend this multimodal framework to demyelinating, neurodegenerative, and traumatic brain conditions, where quantitative MRI metrics may support diagnosis, monitoring of disease progression, and assessment of treatment response. In multiple sclerosis and other demyelinating disorders, SyMRI-derived myelin-related metrics may help characterize tissue changes related to demyelination and repair, whereas IVIM-derived parameters may provide complementary information regarding diffusion and microvascular perfusion. Similar approaches may also be relevant in aging, Alzheimer’s disease, traumatic brain injury, leukodystrophies, and inflammatory white matter disorders, where myelin alteration, axonal injury, neuroinflammation, vascular dysfunction, and extracellular water changes may coexist. In addition, integration of quantitative MRI parameters into radiomics or artificial intelligence-based models may support individualized tissue characterization and future decision-support systems. Because myelin-related tissue properties may also be influenced by activity-dependent neuroplasticity, future studies combining quantitative MRI with functional, cognitive, or molecular markers, such as activity-regulated cytoskeleton-associated protein (Arc/Arg3.1), may provide further insight into the relationship between imaging-derived myelin metrics, neuronal activity, and brain plasticity. To further summarize the translational relevance of the investigated parameters, Table 5 presents the potential biological interpretation and clinical relevance of each quantitative MRI-derived metric.

A key strength of this study is its integrated multimodal design, in which SyMRI-derived myelin-related metrics and IVIM-derived diffusion/perfusion parameters were evaluated within the same healthy adult cohort using consistent ROI placement. This allowed direct cross-modality comparison between quantitative parameters with different physiological bases. In addition, the use of both ROI-averaged WM/GM analysis and ROI-wise regional analysis provided complementary information regarding tissue-level and region-specific associations.

Despite these strengths, several limitations of this work should be considered. First, this study included a relatively small cohort of healthy subjects, which may limit the generalizability of the findings to pathological conditions, particularly demyelinating and neurodegenerative diseases. Second, the age range was relatively broad, and biological variability related to ongoing brain maturation in younger adults or age-related microstructural changes may have influenced quantitative MRI parameters. Third, although participants had no known neurological or psychiatric disorders and structurally normal MRI findings, detailed information regarding chronic systemic diseases, medication use, family history of neurological or psychiatric disorders, and prenatal exposure history was not systematically available. These factors may influence brain tissue properties and should be more comprehensively assessed in future studies. Fourth, the analysis was based on two-dimensional ROI-based measurements rather than whole-brain volumetric segmentation; therefore, global WM and GM volumes were not quantified, and the spatial distribution of myelin-related changes across the entire brain may not have been fully captured. Fifth, although PD showed a strong association with MyCPF in WM, it remains an indirect marker of tissue composition and may be influenced by factors such as water content, inflammation, or edema. Finally, the cross-sectional design precludes assessment of longitudinal changes. Future studies using larger cohorts, disease populations, three-dimensional acquisitions, automated whole-brain segmentation, and longitudinal designs are warranted to validate and extend these findings.

## 5. Conclusions

This study indicates that PD shows a strong and statistically significant inverse association with MyCPF in WM, supporting its potential as a non-invasive marker of myelin-related tissue composition. No significant associations were observed in ROI-averaged GM measures after correction, although region-specific analyses revealed localized effects in subcortical structures, including the caudate and putamen. IVIM-derived parameters showed limited relevance for myelin assessment, likely reflecting their distinct physiological basis related to diffusion and microvascular perfusion rather than intrinsic tissue composition.

## Figures and Tables

**Table 1:** Comparison of ROI-averaged quantitative MRI parameters between GM and WM.

Parameter	GM (Mean ± SD)	WM (Mean ± SD)	*p*-Value	Effect Size (r)	Direction
ADC (×10^−3^ mm^2^/s)	0.726 ± 0.020	0.789 ± 0.027	<0.001	0.88	↑ WM
*D* (×10^−3^ mm^2^/s)	0.703 ± 0.014	0.750 ± 0.028	<0.001	0.85	↑ WM
D* (×10^−3^ mm^2^/s)	0.827 ± 0.060	0.939 ± 0.050	<0.001	0.83	↑ WM
*f*	0.039 ± 0.014	0.053 ± 0.011	<0.001	0.79	↑ WM
T_1_ (ms)	830.7 ± 20.6	561.6 ± 16.9	<0.001	0.92	↑ GM
T_2_ (ms)	77.89 ± 2.16	77.80 ± 2.26	0.901	0.02	≈
PD (%)	79.37 ± 1.99	61.43 ± 1.61	<0.001	0.95	↑ GM

Abbreviations: ADC, apparent diffusion coefficient; *D*, true diffusion coefficient; D*, pseudo-diffusion coefficient; *f*, perfusion fraction; T_1_, longitudinal relaxation time; T_2_, transverse relaxation time; PD, proton density; GM, gray matter; WM, white matter.

**Table 2:** Global brain and myelin-related metrics.

Parameter	Mean ± SD
MyCPF (%)	13.46 ± 0.95
MyC (mL)	183.5 ± 17.89
BPF (%)	87.73 ± 2.90
BPV (mL)	1362.69 ± 78.89
CSF (mL)	191.35 ± 50.42
ICV (mL)	1554 ± 93.16

Abbreviations: MyCPF, myelin-correlated parenchymal fraction; MyC, myelin-correlated volume; BPF, brain parenchymal fraction; BPV, brain parenchymal volume; CSF, cerebrospinal fluid; ICV, intracranial volume.

**Table 3:** Spearman correlations between ROI-averaged WM parameters and myelin metrics.

Parameter (WM)	MyCPF ρ	*p*-Value	q-Value	MyC ρ	*p*-Value	q-Value
ADC (×10^−3^ mm^2^/s)	−0.077	0.707	0.872	−0.050	0.810	0.872
*D* (×10^−3^ mm^2^/s)	0.140	0.495	0.577	0.232	0.255	0.396
D* (×10^−3^ mm^2^/s)	−0.237	0.243	0.397	−0.281	0.165	0.330
*f*	−0.282	0.162	0.330	−0.386	0.051	0.178
T_1_ (ms)	−0.026	0.900	0.900	0.053	0.796	0.810
T_2_ (ms)	0.389	0.050	0.178	0.276	0.172	0.330
**PD** (%)	**−0.736**	**<0.001**	**<0.001** *	−0.435	0.027	0.180

Note: q-values were calculated using the Benjamini–Hochberg false discovery rate (FDR) correction. Statistically significant correlations after correction are indicated by an asterisk (* q < 0.05). Bold values indicate statistically significant correlations after FDR correction. Abbreviations: ρ, Spearman correlation coefficient; MyCPF, myelin-correlated parenchymal fraction; MyC, myelin-correlated volume; ADC, apparent diffusion coefficient; *D*, true diffusion coefficient; D*, pseudo-diffusion coefficient; *f*, perfusion fraction; T_1_, longitudinal relaxation time; T_2_, transverse relaxation time; PD, proton density; WM, white matter.

**Table 4:** Spearman correlations between ROI-averaged GM parameters and myelin metrics.

Parameter (GM)	MyCPF ρ	*p*-Value	q-Value	MyC ρ	*p*-Value	q-Value
ADC (×10^−3^ mm^2^/s)	−0.101	0.624	0.672	−0.345	0.084	0.235
*D* (×10^−3^ mm^2^/s)	−0.009	0.964	0.964	−0.177	0.388	0.603
D* (×10^−3^ mm^2^/s)	−0.156	0.447	0.522	−0.429	0.029	0.203
*f*	−0.264	0.192	0.384	−0.399	0.043	0.201
T_1_ (ms)	0.245	0.227	0.397	0.165	0.421	0.535
T_2_ (ms)	0.267	0.188	0.439	0.170	0.407	0.570
PD (%)	−0.532	0.005	0.073	−0.347	0.083	0.291

Note: q-values were calculated using the Benjamini–Hochberg false discovery rate (FDR) correction. Abbreviations: ρ, Spearman correlation coefficient; MyCPF, myelin-correlated parenchymal fraction; MyC, myelin-correlated volume; ADC, apparent diffusion coefficient; *D*, true diffusion coefficient; D*, pseudo-diffusion coefficient; *f*, perfusion fraction; T_1_, longitudinal relaxation time; T_2_, transverse relaxation time; PD, proton density; GM, gray matter.

**Table 5:** Potential clinical relevance of quantitative MRI-derived parameters in central nervous system disorders.

Parameter	Biological Interpretation	Potential Clinical Relevance
ADC	Overall water diffusivity	May reflect tissue disruption, edema, inflammation, or axonal injury
*D*	True molecular diffusion	May provide diffusion-related information less influenced by perfusion effects
D*	Pseudo-diffusion component	May reflect microvascular perfusion-related signal components
*f*	Perfusion fraction	May provide information related to microvascular perfusion
T_1_	Longitudinal relaxation	Sensitive to tissue composition, water content, and myelin-related changes
T_2_	Transverse relaxation	Sensitive to water content, edema, inflammation, and tissue microstructure
PD	Proton density/mobile proton content	May reflect water/macromolecular composition and MyCPF-related tissue properties in WM
MyCPF	Myelin-correlated parenchymal fraction	May support assessment of myelin-related tissue properties
MyC	Myelin-correlated volume	May provide volumetric information on myelin-related tissue components

## Data Availability

The datasets generated and/or analyzed during the current study are available from the corresponding author upon reasonable request, subject to institutional and ethical restrictions.

## Abbreviations

The following abbreviations are used in this manuscript:

Abbreviation	Definition
ADC	Apparent diffusion coefficient
BPF	Brain parenchymal fraction
BPV	Brain parenchymal volume
CSF	Cerebrospinal fluid
*D*	True diffusion coefficient
*D**	Pseudo-diffusion coefficient
FDR	False discovery rate
*f*	Perfusion fraction
FLIRT	FMRIB’s Linear Image Registration Tool
FSL	FMRIB Software Library
GM	Gray matter
ICV	Intracranial volume
IVIM-DWI	Intravoxel incoherent motion diffusion-weighted imaging
MRI	Magnetic resonance imaging
MyC	Myelin-correlated volume
MyCPF	Myelin-correlated parenchymal fraction
PD	Proton density
ROI	Region of interest
SyMRI	Synthetic MRI
T_1_	Longitudinal relaxation time
T_2_	Transverse relaxation time
WM	White matter

## References

[1] Paus T., Collins D., Evans A., Leonard G., Pike B., Zijdenbos A. (2001). Maturation of white matter in the human brain: A review of magnetic resonance studies. Brain Res. Bull..

[2] Alexander A.L., Hurley S.A., Samsonov A.A., Adluru N., Hosseinbor A.P., Mossahebi P., Tromp D.P., Zakszewski E., Field A.S. (2011). Characterization of Cerebral White Matter Properties Using Quantitative Magnetic Resonance Imaging Stains. Brain Connect..

[3] Yang K., Wu Z., Long J., Li W., Wang X., Hu N., Zhao X., Sun T. (2023). White matter changes in Parkinson’s disease. npj Park. Dis..

[4] Roy-O’Reilly M., Mulavara A., Williams T. (2021). A review of alterations to the brain during spaceflight and the potential relevance to crew in long-duration space exploration. npj Microgravity.

[5] Hagiwara A., Warntjes M., Hori M., Andica C., Nakazawa M., Kumamaru K.K., Abe O., Aoki S. (2017). Symri of the Brain: Rapid Quantification of Relaxation Rates and Proton Density, with Synthetic Mri, Automatic Brain Segmentation, and Myelin Measurement. Investig. Radiol..

[6] Paschoal A.M., Leoni R.F., dos Santos A.C., Paiva F.F. (2018). Intravoxel incoherent motion MRI in neurological and cerebrovascular diseases. NeuroImage Clin..

[7] Lipiński K., Bogorodzki P. (2024). Evaluation of Whole Brain Intravoxel Incoherent Motion (IVIM) Imaging. Diagnostics.

[8] Ji S., Yang D., Lee J., Choi S.H., Kim H., Kang K.M. (2020). Synthetic MRI: Technologies and Applications in Neuroradiology. J. Magn. Reson. Imaging.

[9] Kumar N.M., Fritz B., Stern S.E., Warntjes J.B.M., Chuah Y.M.L., Fritz J. (2018). Synthetic MRI of the Knee: Phantom Validation and Comparison with Conventional MRI. Radiology.

[10] Callaghan M.F., Mohammadi S., Weiskopf N. (2016). Synthetic quantitative MRI through relaxometry modelling. NMR Biomed..

[11] Alghamdi S., Sinclair B., Cowin G., Brereton I., Tesiram Y.A. (2017). Magnetic resonance spin–spin relaxation time estimation in a rat model of fatty liver disease. J. Magn. Reson. Imaging.

[12] Tanenbaum L., Tsiouris A., Johnson A., Naidich T., DeLano M., Melhem E., Quarterman P., Parameswaran S., Shankaranarayanan A., Goyen M. (2017). Synthetic MRI for Clinical Neuroimaging: Results of the Magnetic Resonance Image Compilation (MAGiC) Prospective, Multicenter, Multireader Trial. Am. J. Neuroradiol..

[13] Zou M., Zhou Q., Li R., Hu M., Qian L., Yang Z., Zhao J. (2023). Image quality using synthetic brain MRI: An age-stratified study. Acta Radiol..

[14] Arshad N.H., Abu Hassan H., Nur Farhayu O., Zainudin Z. (2023). Quantifying Myelin in Neonates Using Magnetic Resonance Imaging: A Systematic Literature Review. Clin. Exp. Pediatr..

[15] Alomair O.I., Alghamdi S.A., Abujamea A.H., AlfIfi A.Y., Alashban Y.I., Kurniawan N.D. (2025). Investigating the Role of Intravoxel Incoherent Motion Diffusion-Weighted Imaging in Evaluating Multiple Sclerosis Lesions. Diagnostics.

[16] Federau C., Sumer S., Becce F., Maeder P., O’Brien K., Meuli R., Wintermark M. (2014). Intravoxel incoherent motion perfusion imaging in acute stroke: Initial clinical experience. Neuroradiology.

[17] Le Bihan D., Breton E., Lallemand D., Grenier P., Cabanis E., Laval-Jeantet M. (1986). MR imaging of intravoxel incoherent motions: Application to diffusion and perfusion in neurologic disorders. Radiology.

[18] Finkenstaedt T., Klarhoefer M., Eberhardt C., Becker A.S., Andreisek G., Boss A., Rossi C. (2017). The Ivim Signal in the Healthy Cerebral Gray Matter: A Play of Spherical and Non-Spherical Components. Neuroimage.

[19] Granberg T., Uppman M., Hashim F., Cananau C., Nordin L., Shams S., Berglund J., Forslin Y., Aspelin P., Fredrikson S. (2016). Clinical Feasibility of Synthetic MRI in Multiple Sclerosis: A Diagnostic and Volumetric Validation Study. Am. J. Neuroradiol..

[20] Piredda G.F., Hilbert T., Thiran J., Kober T. (2020). Probing myelin content of the human brain with MRI: A review. Magn. Reson. Med..

[21] Zhang H., Hu L., Qin F., Chang J., Zhong Y., Dou W., Hu S., Wang P. (2024). Synthetic MRI and diffusion-weighted imaging for differentiating nasopharyngeal lymphoma from nasopharyngeal carcinoma: Combination with morphological features. Br. J. Radiol..

[22] Alomair O.I., Alghamdi S.A., Abujamea A.H., Aljarallah S., Alkhawajah N.M., Alshuhri M.S., Alashban Y.I., Kurniawan N.D. (2025). The Utility of Intravoxel Incoherent Motion Metrics in Assessing Disability in Relapsing–Remitting Multiple Sclerosis. Diagnostics.

[23] Alomair O.I., Alshuhri M.S., Al-Mubarak H.F., Alghamdi S.A., Abujamea A.H., Aljarallah S., Alkhawajah N.M., Alashban Y.I., Kurniawan N.D. (2025). IVIM-DWI-Based Radiomics for Lesion Phenotyping and Clinical Status Prediction in Relapsing–Remitting Multiple Sclerosis. J. Clin. Med..

[24] Lange F.J., Arthofer C., Bartsch A., Douaud G., McCarthy P., Smith S.M., Andersson J.L.R. (2024). MMORF—FSL’s MultiMOdal Registration Framework. Imaging Neurosci..

[25] Jenkinson M., Beckmann C.F., Behrens T.E.J., Woolrich M.W., Smith S.M. (2012). FSL. Neuroimage.

[26] Yushkevich P.A., Piven J., Hazlett H.C., Smith R.G., Ho S., Gee J.C., Gerig G. (2006). User-guided 3D active contour segmentation of anatomical structures: Significantly improved efficiency and reliability. NeuroImage.

[27] Wansapura J.P., Holland S.K., Dunn R.S., Ball W.S. (1999). NMR relaxation times in the human brain at 3.0 tesla. J. Magn. Reson. Imaging.

[28] Warntjes J., Dahlqvist O., Lundberg P. (2007). Novel method for rapid, simultaneous *T*_1_, *T^*^*_2_, and proton density quantification. Magn. Reson. Med..

[29] Lazari A., Lipp I. (2021). Can MRI measure myelin? Systematic review, qualitative assessment, and meta-analysis of studies validating microstructural imaging with myelin histology. NeuroImage.

[30] Le Bihan D., Iima M., Federau C., Sigmund E.E. (2018). Intravoxel Incoherent Motion (IVIM) MRI: Principles and Applications.

[31] Beaulieu C. (2002). The basis of anisotropic water diffusion in the nervous system—A technical review. NMR Biomed..

[32] Jelescu I.O., Budde M.D. (2017). Design and Validation of Diffusion MRI Models of White Matter. Front. Phys..

[33] Weiskopf N., Suckling J., Williams G., Correia M.M., Inkster B., Tait R., Ooi C., Bullmore E.T., Lutti A. (2013). Quantitative multi-parameter mapping of R1, PD*, MT, and R2* at 3T: A multi-center validation. Front. Neurosci..

[34] Hagiwara A., Hori M., Yokoyama K., Takemura M., Andica C., Tabata T., Kamagata K., Suzuki M., Kumamaru K., Nakazawa M. (2016). Synthetic MRI in the Detection of Multiple Sclerosis Plaques. Am. J. Neuroradiol..

[35] Drori E., Cohen L., Arkadir D., Yahalom G., Mezer A.A. (2025). Multiparametric quantitative MRI uncovers putamen microstructural changes in Parkinson’s disease. npj Park. Dis..

[36] Sbaihat H., Roenneke K., Müller D., Ladopoulos T., Schneider R., Krieger B., Bellenberg B., Lukas C. (2025). MRI-based human brain atlases of R1, R2, proton density, and myelin volume fraction using synthetic quantitative imaging at 1.5 T. J. Neurol..

[37] Campbell J.S., Leppert I.R., Narayanan S., Boudreau M., Duval T., Cohen-Adad J., Pike G.B., Stikov N. (2018). Promise and pitfalls of g-ratio estimation with MRI. Neuroimage.

[38] Coelho S., Baete S.H., Lemberskiy G., Ades-Aron B., Barrol G., Veraart J., Novikov D.S., Fieremans E. (2022). Reproducibility of the Standard Model of diffusion in white matter on clinical MRI systems. NeuroImage.

[39] Leutritz T., Seif M., Helms G., Samson R.S., Curt A., Freund P., Weiskopf N. (2020). Multiparameter mapping of relaxation (R1, R2*), proton density and magnetization transfer saturation at 3 T: A multicenter dual-vendor reproducibility and repeatability study. Hum. Brain Mapp..

